# Supplementary material for: Discovery and application of insertion-deletion (INDEL) polymorphisms for QTL mapping of early life-history traits in Atlantic salmon
Source: BMC Genomics. 2010 Mar 8;11:156. doi: 10.1186/1471-2164-11-156 (PMC2838853; doi:10.1186/1471-2164-11-156)
Supplement: Additional file 2 — Information on developed 76 locus single-run INDEL panel in Atlantic salmon. Information on fluorescence labeling, primer concentrations, PCR pooling and links to alignments, INDEL motifs and GENESCAN (Burge and Karlin 1997) predictions of genes/exons are available in html format. [file 1471-2164-11-156-S2.ZIP › Additionalfile2/snpsummary8229.html]

```
Cluster 2546 Contig 3

prev  Summary    Contig List  next
```

Size of Consensus sequence = 768

Number of sequences = 6

Minimum redundancy = 2

Key

A gi|29327444|gb|CB516218.1|CB516218 ssalrgb554290 mixed\_tissue Salmo salar cDNA, mRNA sequence  
B gi|24391306|gb|CA061063.1|CA061063 ssalrgb529027 mixed\_tissue Salmo salar cDNA, mRNA sequence  
C gi|24384640|gb|CA054397.1|CA054397 ssalrgb517233 mixed\_tissue Salmo salar cDNA, mRNA sequence  
D gi|89865048|gb|DY721171.1|DY721171 EST\_ssal\_rgb2\_76910 ssalrgb2 mixed\_tissue Salmo salar cDNA Salmo salar cDNA clone ssal\_rgb2\_626\_125\_fwd 3', mRNA sequence  
E gi|89837789|gb|DY696829.1|DY696829 EST\_ssal\_rgb2\_52568 ssalrgb2 mixed\_tissue Salmo salar cDNA Salmo salar cDNA clone ssal\_rgb2\_585\_019\_fwd 3', mRNA sequence  
F gi|117513316|gb|EG845075.1|EG845075 EST\_ssal\_eve\_37861 ssaleve thyroid Salmo salar cDNA Salmo salar cDNA clone ssal\_eve\_551\_210\_rev 5', mRNA sequence

5 SNPs detected

A B C D E F  cosegregation weighted

130 G G - - G .   5/5 83.33
131 A A - - A .   5/5 83.33
132 G G - - G .   5/5 83.33
133 G G - - G .   5/5 83.33
134 T T - - T .   5/5 83.33
